# Painful intervertebral disc degeneration and inflammation: from laboratory evidence to clinical interventions

**DOI:** 10.1038/s41413-020-00125-x

**Published:** 2021-01-29

**Authors:** Feng-Juan Lyu, Haowen Cui, Hehai Pan, Kenneth MC Cheung, Xu Cao, James C. Iatridis, Zhaomin Zheng

**Affiliations:** 1 School of Medicine, South China University of Technology, Guangzhou, China; 2 Department of Spine Surgery, The First Affiliated Hospital, Sun Yat-Sen University, Guangzhou, China; 3 Guangdong Provincial Key Laboratory of Malignant Tumor Epigenetics and Gene Regulation, Sun Yat-sen Memorial Hospital, Sun Yat-sen University, Guangzhou, China; 4 Breast Tumor Center, Sun Yat-sen Memorial Hospital, Sun Yat-sen University, Guangzhou, China; 5 Department of Orthopedics & Traumatology, The University of Hong Kong, Hong Kong, SAR China; 6 Department of Orthopedic Surgery, Johns Hopkins University, Baltimore, MD USA; 7 Leni and Peter W. May Department of Orthopedics, Icahn School of Medicine at Mount Sinai, New York, NY USA; 8 Pain Research Center, Sun Yat-sen University, Guangzhou, China

**Keywords:** Pathogenesis, Bone

## Abstract

Low back pain (LBP), as a leading cause of disability, is a common musculoskeletal disorder that results in major social and economic burdens. Recent research has identified inflammation and related signaling pathways as important factors in the onset and progression of disc degeneration, a significant contributor to LBP. Inflammatory mediators also play an indispensable role in discogenic LBP. The suppression of LBP is a primary goal of clinical practice but has not received enough attention in disc research studies. Here, an overview of the advances in inflammation-related pain in disc degeneration is provided, with a discussion on the role of inflammation in IVD degeneration and pain induction. Puncture models, mechanical models, and spontaneous models as the main animal models to study painful disc degeneration are discussed, and the underlying signaling pathways are summarized. Furthermore, potential drug candidates, either under laboratory investigation or undergoing clinical trials, to suppress discogenic LBP by eliminating inflammation are explored. We hope to attract more research interest to address inflammation and pain in IDD and contribute to promoting more translational research.

## Introduction

Low back pain (LBP) is a common clinical symptom occurring predominantly in middle to old age.^1^ In 2015, the prevalence of LBP in adults worldwide was 7.3%. From 1990 to 2015, the number of people with disabilities due to LBP increased by 54%.^2^ In total, ~40% of the population suffers from LBP during their lifetime.^3^ This condition is now the number one cause of the world’s disability burden.^4^ Recurrent LBP impairs the patient’s physical and mental health and places a heavy burden on health care and social support systems.^5^

Intervertebral discs (IVDs) are complex fibrocartilaginous tissues that connect adjacent vertebral bodies to enable spinal motion. IVD degeneration (IDD) increases with age, with more than 80% of IVDs exhibiting degeneration-related changes in people over 50 years old.^6^ IDD is a widely recognized cause of back pain.^7,8^ During IDD, IVD cells exhibit increased proinflammatory cytokines.^7,8^ Degeneration also results in degradation of the extracellular matrix and loss of hydrophilic matrix molecules, which can lead to structural and biomechanical changes^9^ and is a leading cause of increased inflammation, nerve ingrowth^10,11^ and release of pain factors.^12^

IDD is a complex process. Proinflammatory conditions may be a critical factor in IDD.^7,8^ Recent research has identified inflammatory mediators and signaling pathways as important factors in the onset and progression of disc degeneration.^13^ Inflammation mediated by immune cells was enhanced in degenerated IVDs, and degradation products of these cells, such as IL-4, IL-6, IL-12, IFN-γ, and MMPs, led to a reduction in NP cell number and deterioration of the IVD microenvironment.^14^ Long-term inflammation recruits inflammatory cells, which further exacerbate this situation.^15^ Furthermore, inflammatory mediators such as TNF-α and IL-1β induce the expression of pain-related factors such as nitric oxide (NO), cyclooxygenase 2 (COX-2), and nerve growth factors (NGF), which promote nerve ingrowth.^12^ All of these factors together contribute to the occurrence of LBP.

Treating discogenic LBP requires a good understanding of IDD and the underlying inflammation. Similarly, basic and translational science studies are most potent when they consider treatments for IDD, including their effects on pain relief. Historically, laboratory research has often focused on the biological repair of injured or degenerated IVDs with less attention given to pain suppression. However, clinical research has often emphasized the urgent need of patients for pain relief without sufficient attention to how these approaches can slow IDD or promote healing. This gap between clinical and basic research is receiving increased attention and narrowing, while researchers take a more holistic approach to science and patient care in their study designs.

In this review, we tried to provide an overview of discogenic LBP studies with a focus on advances in inflammation-related pain in IDD. We review the role of inflammation in IDD and pain induction, as well as the currently available animal models for the study of painful IDD and the underlying signaling pathways contributing to inflammation and discogenic pain production. In addition, we explored laboratory and clinical pain elimination candidates and trials under development. Overall, we hope this review contributes to filling the gap between laboratories and clinics by clarifying the current understanding of how pain is produced in disc degeneration and inflammation, what kind of animal models we can use to study discogenic pain, and what strategies we are currently developing.

## Disc degeneration is an important cause of LBP

### Structure of healthy IVDs

Healthy IVDs are a three-part complex consisting of a gelatinous proteoglycan-rich nucleus pulposus (NP) in the center, the interwoven collagenous layers of annulus fibrosus (AF) on its periphery, and cartilaginous and vertebral endplates (EP) on the superior and inferior surfaces. The NP is subjected to high pressure, which is resisted by the substantial hoop stress of the AF to prevent the outward expansion of the NP. AF resists large tensile and compressive strains when a torsional strength is applied to the disc. Under normal conditions, NP and AF work together to provide the mechanical properties of the IVD to maintain a high-stress load on the vertebral body.

### Degenerative IVD

**Fig. 1:**
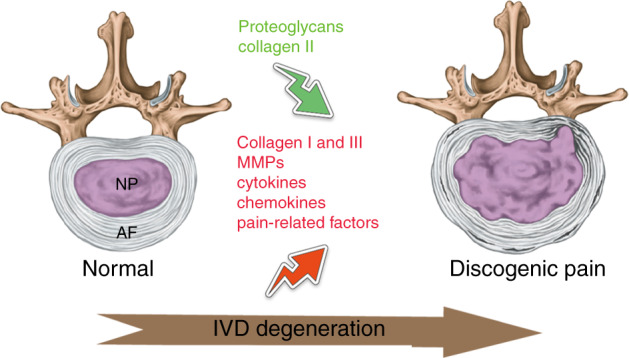
Illustration of degenerative changes in painful intervertebral discs. During IVD degeneration, proteoglycans and collagen II are decreased, while collagen I and III are increased in the extracellular matrix. Emerging cytokines, chemokines, and pain-related factors synergistically contribute to discogenic pain development. IVD intervertebral disc, NP nucleus pulposus, AF annulus fibrosus

The etiology of IDD is complicated. Most degeneration begins in adults and progresses with age.^16^ The large vacuolated notochordal-like cells in the NP start to disappear at an earlier stage of life at ~10 years of age, which some consider to be the initiation of the degenerative process.^17^ Although endogenous progenitor cells have been found in the disc,^18^ indicating the potential for self-repair, there is a lack of evidence of spontaneous regeneration in the human IVD. Consequently, the synthesis of proteoglycans is reduced, accompanied by the conversion of collagen synthesis, in which type II collagen is reduced and type I and type III collagen are increased (Fig. 1).^19^ In addition, the synthesis/activity of matrix metalloproteinase (MMP) is increased.^20^ Apoptotic NP cells are also significantly increased.^21^ Calcification of the cartilage endplates results in a reduced supply of nutrients to the intervertebral disc,^22^ further exacerbating the process. EP sclerosis is also reported with EP calcification and ossification caused by aberrant mechanical loading.^23^ Degradation of the extracellular matrix (ECM) and loss of proteoglycans result in a decreased loading capacity and height of the IVD.^7^ The high osmotic pressure and acidic environment in the IVD further exacerbate the stress state.^21^

### Degenerative IVD and discogenic pain

Discogenic pain refers to pain induced by degenerative changes in the IVD, resulting in the early stages of disc degeneration.^24^ Numerous studies have confirmed that LBP and IDD are closely connected. Arnbak et al. performed an analysis of 1037 patients with persistent LBP, confirming that the LBP incidence increased with the degree of degeneration.^25^ Middendorp et al. found that the Oswestry Disability Index (ODI) for patients with LBP also correlates with disc degeneration grade.^26^ Similarly, an increased IVD Pfirrmann grade was accompanied by an increase in ODI in patients.^26^ In one study, 87% of patients with persistent LBP had disc degeneration.^27^ Pain-related molecules, including tumor necrosis factor (TNF) alpha, interleukin (IL)-1 beta, IL-4, IL-6, IL-8, IL-12, prostaglandin E2 (PGE2), interferon-gamma, and nitric oxide (NO), were found to be upregulated in herniated human IVDs.^8,28^

In healthy spines, only the outer AF is innervated.^29^ Animal models and human clinical specimens have shown sensory innervation in the lumbar disc and sensory nerve ingrowth to the inner layer of the disc,^30–32^ which is an important cause of pain.^29,33^ In degenerated and herniated discs, nerves were found to be localized in distorted tissues.^34^ In a spontaneously degenerating mouse model, age-dependent increases in sensory innervation were found in the IVDs.^35^ Increasing innervation was also found in vertebral endplates from discogenic LBP patients with modic changes.^36^ The identified inducers of nerve ingrowth include TNF-α, IL-1,^37–39^ and NGF.^33^ The discogenic pain signal passes through the IVD and adjacent structures and is transmitted through peripheral afferent nerve fibers.^29^ The cells are located in the DRG and projected by projection neurons in the spinal dorsal horn to the brain region. Analysis of the role of the peripheral nervous system during painful IDD has become an essential area of research.^29,40–42^ Nerve fibers interact with inflammatory mediators in the NP and cause discogenic LBP.^30^ Compared with herniated IVDs, painful discs contain more inflammatory mediators.^43^

However, not all patients with disc degeneration are symptomatic^44^ with LBP.^45^ Studies have shown that the proportion of severely degraded but painless IVDs increases with age.^26,46^ The lack of persistent inflammation might be crucial to determine whether a degenerated disc becomes symptomatic.^47^ Taken together, these reports strongly suggest a correlation between sensory nerve ingrowth, inflammatory mediators, and discogenic LBP.

## Animal models of painful IVD degeneration

Animal experiments are indispensable for studying painful disc degeneration and inflammation. The establishment of an animal model of experimental IDD can provide experimental methods for studying painful IDD. Ideally, among all species, primates are ideal for studying LBP, as they are the species closest to humans. Other large animal models include pigs,^48^ sheep,^49^ goats,^50^ and dogs.^51^ Rodent models are the most widely used animal model for studying LBP.^42^

**Fig. 2:**
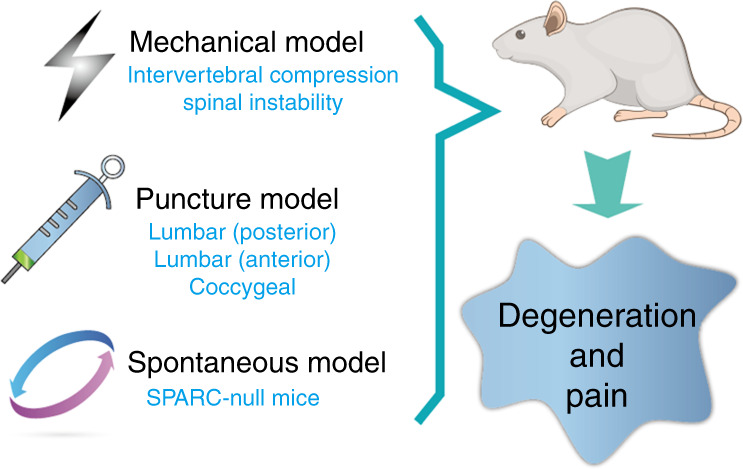
Animal models of painful IVD degeneration. These models include a mechanical model, a needle puncture model through a posterior or anterior approach, and a spontaneous IVD degeneration and LBP model represented by SPARC-null mice. IVD intervertebral disc, LBP low back pain, SPARC secreted protein acidic and rich in cysteine

Here, we discuss available animal models of IDD with documented pain symptoms. These animal models can be classified into three categories (Fig. 2): (1) needle puncture models, (2) mechanical models that apply abnormal mechanical stress to the IVD, and (3) spontaneous IDD models.

### IVD puncture models

The disc puncture models involve performing puncture through the posterior or anterior direction. Olmarker punctured lumbar IVDs from the posterior direction with a 0.4 mm diameter needle in rats.^52^ Posterior puncture could induce spontaneous pain behavior, such as increased “grooming” and “wet-dog shakes”.^52^ In this model, the left facet joint was removed, and the structure of the posterior column was destroyed to some extent. The resulting disc degeneration and pain may contribute to spinal instability. To explain the mechanism of this behavioral change, Nilsson et al. compared rats that received superficial disc injury without NP leakage and healthy NP. The study showed that the NP leakage effects were more pronounced than the effects induced by disc injury.^53^

Instead of posterior puncture, Kim et al. used anterior surgery to remove the rat NP through the ventral aspect of abdomens. This process led to inflammatory cell infiltration, proteoglycan loss, and intervertebral height reduction. Nine weeks after the surgery, pain behavior represented by pressure hyperalgesia developed over the lower back.^54^ Lee et al. studied IVD degenerative changes with pain development after a 10 mL CFA (complete Freund’s adjuvant) injection into adult rat lumbar IVDs through an anterior approach. A significant increase in pain, assessed by the hind paw withdrawal response, occurred at 7 weeks postoperatively, accompanied by increased calcitonin gene-related peptide (CGRP) and inducible nitric oxide synthase (iNOS) expression in the DRG.^55^ Lai et al. further modified the anterior model by intradiscal injection of saline into rat lumbar IVDs, which resulted in increased pain, as evaluated by increased grooming duration, decreased mechanical withdrawal thresholds, and decreased thermal withdrawal latency.^56^ Later, both Lai et al.^57^ and Evashwick‐Rogler et al.^39^ punctured lumbar IVDs anteriorly, and found that TNF-α injection had a greater and more consistent increase in pain, while an anti-TNF-α antibody alleviated pain to sham levels. The pain threshold was also found to be linearly associated with IDD and intradiscal TNF-α expression^39^ and substance P expression in the DRG.^57^ These results indicated that anterior disc injection of TNF-α is a useful painful disc degeneration model. Millecamps also induced LBP in mice by lumbar anterior disc puncture^58^ and observed the development of pain behavioral signs such as tail suspension and grip force, radiating hypersensitivity, and motor impairment, accompanied by increased dorsal innervation and reduced disc height.^58^

In addition to that of lumbar discs, puncture at the coccygeal disc level can induce pain behavior. Isma described a novel rat coccygeal model of pain induced by a puncture at levels C5–C6 and/or C4–C5.^59^ Both thermal hyperalgesia and mechanical allodynia, which were detected by applying stimuli to the tail’s ventral base, were found to evoke puncture-induced disc formation in the rat tail. Thermal hypoalgesia-induced changes were also observed in the ventral middle part of the tail through tail-flick testing.^59^

The onset of inflammation might play an important role in pain development in these puncture models. Lee et al. found that pathological innervation lasted up to 12 months in the anterior puncture mouse model, while macrophage infiltration started from day 4 post surgery both dorsally and ventrally.^60^ Most importantly, the large amount of inflammatory mediators behind the disc chemically and biologically stimulate spinal nerve roots.^7^ In another study, no pain behavior changes were detected with anterior disc puncture, whereas posterior disc puncture resulted in mechanical allodynia lasting from 1 day to 21 days postoperatively.^61^ Interestingly, in this study, increased inflammatory cytokines were detected in the DRG after posterior but not anterior puncture,^61^ which indicates that inflammatory factors are indispensable for producing pain.

### Mechanical animal model

Mechanical animal models induce disc degeneration by altering the normal biomechanical state. These models include the hindlimb unloading model,^62^ bipedal animal model,^63^ caudal spine mechanical animal model,^64–66^ tail bend model,^67^ and spine instability model.^68^ Among these models, only the caudal spine mechanical and spine instability models have been demonstrated to simulate LBP.

For the caudal spine mechanical models, pain-associated molecule expression was investigated instead of pain behavior changes. Chubinskaya et al. used a vertebral compression rat model to study the relationship between stress and pain.^69^ Two 0.8 mm-diameter Kirschner’s wires were inserted percutaneously through the third and fifth coccygeal vertebrae. Each wire was fixed separately to a specially designed aluminum ring consisting of two 30 mm diameter external rings. Two rings were linked with four rods to immobilize and chronically compress Kirschner’s wires until the tail offered maximum angular deformity. The compressed IVD showed signs of degeneration and inflammation, including the appearance of MMP-13, TNF-alpha, and IL-1 beta, as well as the expression of pain-related molecular markers, such as substance P.^69^ Using coccygeal IVD compression devices, Moyagi et al. demonstrated that disc compression in rats produces a long-lasting increase in inflammatory mediators in IVDs and neuropeptides, such as CGRP and growth-associated phosphoprotein 43, in DRGs.^70^ Moreover, disc compression induced persistent expression of activating transcription factor 3a, a nerve injury marker, and regeneration of the afferent fibers innervating IVDs.^70^ Suzuki et al. also found sustained upregulation of CGRP in DRG neurons in osteoporotic vertebrae after compression.^71^

The spinal instability model was first established by disrupting the spine structure. A mouse spondylosis model was prepared via surgical resection of the posterior spinal element. Ariga et al. observed a large amount of cell apoptosis and destruction of EP in the IVD of the spondylosis model compared with naturally aged IVD.^68^ Fukui et al. established a rat spinal instability model by completely resecting the bilateral L4–L5 facet joints. IDD, LBP, and neuropathic pain accompanying gait abnormalities were observed in this model after 7 weeks.^72,73^ Bian and Zheng et al.^74,75^ resected the lumbar 3rd to 5th (L3–L5) spinous processes along with the supraspinous and interspinous ligaments to promote instability of the mouse lumbar spine, which produced stable degeneration without significant kyphosis.^74,76^ In this model, pain was found to be caused by netrin-1^+^ osteoclasts that initiate porosity of endplates and sensory innervation.^77^

### Spontaneous disc degeneration model

Secreted protein acidic and rich in cysteine (SPARC) is a matricellular protein involved in collagen deposition, cell–ECM interactions, and ECM remodeling.^78^ IVD cells of elderly subjects with disc degeneration had reduced SPARC expression.^79^ SPARC-null mice showed defective connective tissue^80^ and accelerated disc degeneration.^80,81^ More importantly, SPARC-null mice displayed behavioral signs indicating chronic low back and radicular pain.^82^ The development of behavioral signs of axial and radiating LBP and reduced physical function increased with aging in SPARC-null mice.^83^ Interestingly, SPARC-null mice displayed cold hypersensitivity and avoidance of stretching along the spine’s axis but were not mechanically or thermally hypersensitive. Moreover, the sensitivity was site-specific; the hind paw and back, but not the tail, were cold sensitive. Furthermore, icilin-evoked behavior increased subsequent applications to the hind paw but not the lip.^84^

### Summary of the above animal models

All the above mentioned animal models are similar in that they can cause disc degeneration with measurable pain development. Due to the different methods adopted in these models to compromise the IVD, they have unique advantages and disadvantages, as summarized in Table 1. IVD puncture models are established through posterior or anterior injury of the disc, and they have the advantage of ease of establishment despite some drawbacks. Posterior disc puncture will inevitably damage the posterior spinal structure. Resection of the spinous processes and lamina can cause kyphosis in animals. In addition, the physical–mechanical puncture itself has a risk of irritating the spinal cord and nerve roots, which will bias the study. The caudal spine mechanical models mimic the natural development of human LBP by applying abnormal compression, therefore representing a more natural procedure of pain development. However, the establishment of these models is complex and requires skillful techniques. It will be difficult to universally adopt this model. The spine instability models take advantage of the abnormal stress generated by the unstable spine on the IVD; however, the effect of this stress may cause excessive destruction of the posterior column and lead to severe kyphosis.^23^ Therefore, the scope of spine injury must be carefully designed. As a spontaneous disc degeneration model, SPARC-null mice provide a convenient and controllable model to study spontaneous disc degeneration and pain.^41,85^ However, these mice also have some shortcomings as a genetically modified model. The lack of SPARC may affect IVD in multiple ways in addition to what we already know. Conversely, SPARC deficiency may not be fully responsible for IDD, and pain occurs under natural conditions.

**Table 1:** Comparison of animal models for painful disc degeneration

Animal models	Degenerative mechanisms	Advantages	Disadvantages
Disc puncture models	By posterior or anterior injury of the IVD	Easy to establish	Need to damage posterior spinal structure; risk of irritating spinal cord and nerve roots
Mechanical animal model	By applying abnormal compression to the IVD	Mimic the natural development of human LBP	Establishment is complex. Need to carefully control the degree of compression to avoid excessive damage
Spontaneous disc degeneration model	By the abnormal expression of a target gene	Convenient and controllable	Unnatural, only represents a small proportion of painful disc degeneration

## The association of inflammation with painful IVD degeneration

### Inflammatory molecules are increased in and further promote IVD degeneration

Increased levels of inflammatory molecules have been found in degenerated IVDs. TNF-α expression was found to be elevated in the IVD and peripheral serum in patients with degenerated IVDs.^86^ Serological analysis of 392 populations demonstrated that IL-6 was increased in patient serum^87^ with IDD as well as in degenerated IVDs^88^ compared to that of healthy controls. Degenerated and herniated IVDs contained increased IL-17 expression and Th17 lymphocyte infiltration.^28^ Gruber et al. also showed elevated IL-17 expression in degenerated human discs and increased production of IL-17 by disc cells under IL-1β and TNF-α stimulation.^89^

Furthermore, inflammatory molecules have been found to initiate or accelerate IDD. The presence of proinflammatory cytokines promotes the degenerative process, thereby exacerbating degenerative symptoms in IVDs.^8,90^ Denaturation is thought to be partially mediated by infiltrating inflammatory cells and further aggravates disc degeneration.^91,92^ Proinflammatory mediators, including TNF-α, IL-1α/β, IL-2, IL-4, IL-6, IL-8, IL-10, IL-17, IFN-γ, chemokines, and prostaglandin (PGE) 2,^7,8,29^ may also influence the autophagy, senescence, and apoptosis of disc cells.^93–96^ As the key inflammatory mediators of disc degeneration, TNF-α and IL-1β can induce disc degeneration by decreasing anabolic ECM proteins, such as aggrecan and collagen II, and promoting catabolic enzymes, such as a disintegrin and metalloproteinase with thrombospondin motifs (ADAMTS)-4 and -5 and matrix metalloproteinases (MMPs)-1, -2, -3, -4, -13, and -14.^97–105^ TNF-α suppresses the production of ECM, including collagen, aggrecan, and fibromodulin, but increases the expression of MMP-3, -9, and -13 and NGF.^106^ TNF-α and IL-1β also regulate chemokine (C-C motif) ligand 3 (CCL3) expression in NP cells and promote macrophage infiltration through the CCL3-CCR1 axis in degenerated herniated discs.^92^ TNF-α overexpression in mice resulted in early onset spontaneous IVD herniation without affecting collagen and aggrecan expression in the NP and AF.^107^ Global IL-1α/β knockout in mice resulted in a more degenerative phenotype in the AF and collagen type and maturity changes, accompanied by alterations in systemic cytokine levels and vertebral bone morphology.^108^

In addition, IFN-γ and IL-17 synergistically promote the release of inflammatory mediators in disc cells.^109^ IL-6 and IL-6 soluble receptors synergistically potentiate the catabolic effects of IL-1β and TNF-α on human NP cells, along with COX-2 and PGE-2 levels.^110^

In summary, inflammatory cytokines are elevated in degenerated IVDs, and when used as stimuli, they could cause or exacerbate disc degeneration. Therefore, inflammation plays an important role in the degeneration of IVDs.

### Inflammatory molecules are increased in and can cause discogenic pain

Innervation is thought to be an important process in the production of discogenic pain. IVD tissue is generally thought to contain no neural structure, but studies have found that the outer annular layer of the healthy IVD has nerve fibers that rarely extend into the inner AF and NP.^111,112^ The normal IVD is primarily innervated by small DRG neurons consisting of NGF-dependent and glial cell line-derived neurotrophic factor (GDNF)-dependent neurons, which express high-affinity NGF and the brain-derived neurotrophic factor (BDNF) receptors TrkA, TrkB, and GDNF.^30^ Patients with chronic LBP have abundant peripheral nerve fibers in the NP and inner AF.^29,30,32^ During degeneration of the IVD, the nerve fibers gradually extend to the inner layer of the AF and even the NP, accompanied by the expression of pain transmitter substance P,^32^ which is highly relevant to the genesis of discogenic LBP.^29,30,32^

It has been proposed that the expression of inflammatory cytokines is increased in painful IVDs.^7,113^ The expression of IL-6, IL-8, and PGE2 is increased in IVD samples from patients with LBP.^114^ IL-1β and IL-6 levels are enhanced in painful herniated discs.^115^ Ingrowth of vascularized granulation tissue along tears was reported, extending from the outer AF to the NP in patients with discogenic LBP, accompanied by abundant macrophage and mast cell infiltration.^116^ Posterior rupture of AF stimulated TNF-α and IL-1β expression, thus inducing the DRG inflammatory response and mechanical hyperalgesia in a rat model.^61^ Elevated expression of MMP-10, NGF and substance P was found in painful degenerated discs.^117^ These studies indicate that painful discs contain increased levels of proinflammatory mediators.

However, inflammatory factors can cause discogenic pain. Studies have shown that the neurotrophic factors NGF and BDNF contribute to both innervation of degenerating discs and neuronal sensitization^10,118,119^ in mature peripheral afferent fibers and result in the development of chronic pain.^120^ A direct relationship between nociceptive nerve ingrowth and NGF production by blood microvessels was found in painful IVDs.^121^ While degenerative IVDs can secrete many inflammatory and pain mediators, including TNF-α, IL-1β, IL-6, IL-8, substance P, and prostaglandin E2, these factors not only reduce proteoglycan and collagen synthesis but also stimulate nerve fiber ingrowth and induce pain.^7^ Safieh-Garabedian et al. showed that IL-1β promoted NGF expression during inflammation.^122^ Abe et al. demonstrated that IL-1β and TNF-α stimulated the production of NGF by human IVD cells.^123^ Lai et al. reported that intradiscal TNF-α injection led to more painful behavior and stimulated substance P expression in DRGs compared to saline control.^57^ Purmessur et al. also reported that IL-1β significantly increased the expression of NGF and BDNF, while TNF-α upregulated substance P in human NP cells.^124^ Lee et al. found that IL-1β generated during IDD further stimulated VEGF, NGF, and BDNF production and induced angiogenesis and innervation in degenerative IVDs.^125^ Gruber et al. showed that IL-1β significantly elevated BDNF, neurotrophin 3, neuropilin 2, and NGF expression in annulus cells.^126^ Clinically, COX-2 inhibitors, which in turn inhibit PGE2 synthesis, could significantly alleviate LBP.^127^

All these studies suggest that inflammatory cytokines play a major role in the production of NGF, BDNF, and neurotrophins, which results in nerve ingrowth into the disc and thus generates discogenic pain.

## Signaling pathways involved in painful IVD degeneration

**Fig. 3:**
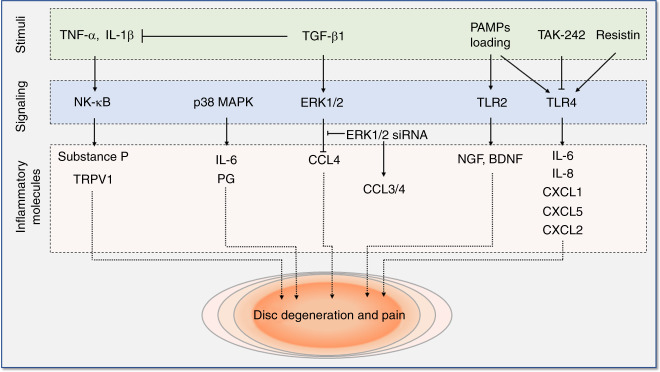
Signaling pathways involved in intervertebral disc degeneration and pain. These pathways include NF-κB signaling, MAPK signaling, and TLR signaling. NF-κB nuclear factor kappa-B, MAPK mitogen-activated protein kinase, TLR Toll-like receptor. IL interleukin, TRPV1 transient receptor potential cation channel subfamily V member 1, PG prostaglandins, TGF-β1 transforming growth factor β1, ERK extracellular signal-regulated kinases, CCL chemokine (C-C motif) ligand, PAMP pathogen-associated molecular patterns, NGF nerve growth factor, BDNF brain-derived neurotrophic factor, CXCL chemokine (C-X-C motif) ligand

Various pathways, such as the nuclear factor kappa-B (NF-κB) and mitogen-activated protein kinase (MAPK) pathways, have been found to play essential roles in the production of inflammatory factors and catabolism of ECM in the IVD,^128^ and increasing evidence supports the importance of these signaling pathways and Toll-like receptor (TLR) signaling in painful disc diseases, as summarized in Fig. 3.^41,129–132^

### NF-κB signaling

NF-κB is a canonical downstream pathway of inflammatory cytokines, such as TNF-α and IL-1β.^133^ NF-κB activity is physically inhibited when an inhibitor of kappa B (IκB) binds to p65. Once stimulated, p65 is rapidly released from the NF-κB heterodimer and IκB and translocates to the nucleus, where it activates the transcription of target genes. Numerous studies have shown that the NF-κB signaling pathway plays a major role in inflammatory cytokine production and secretion,^128^ which is closely associated with disc degeneration and the genesis of discogenic pain.

Recently, it was reported that *Propionibacterium acnes* induces substance P and CGRP expression in the IVD and obvious LBP symptoms by stimulating NP cells to secrete the pro-algesic factor IL-8/CINC-1 through the TLR2-NF-κB p65 pathway, which may provide a promising alternative therapeutic strategy for LBP patients in the clinic.^129^ Moreover, Ahmed et al. found that NF-κB activation correlates with the expression of substance P and transient receptor potential cation channel subfamily V member 1 (TRPV1) in IVD tissues and may be associated with the generation or maintenance of peripheral pain by regulating pain-related neuropeptides in patients with degenerative disc diseases.^130^ Walter et al. similarly identified a relationship between TRPV channels and catabolism and found that TNF-α sensitizes IVD cells to induce a proinflammatory and catabolic phenotype under load via TRPV4 signaling.^134^ These new studies indicated that the NF-κB signaling pathway is not only implicated in inflammatory cytokine secretion but also plays a vital role in discogenic pain production.

### MAPK signaling

MAPKs are a family of highly conserved pathways, including three major subfamilies, the extracellular signal-regulated kinases (ERK), c-Jun NH2-terminal kinase (JNK), and p38 isoforms,^135^ allowing the cells to respond to multiple extracellular stimuli, such as hormones, growth factors, inflammatory cytokines, and other stresses.^136^ MAPKs are master regulators of inflammatory responses. A study from Kim et al. showed that p38 MAPK is involved in IL-6 and prostaglandin (PG) secretion from AF cells when cocultured with macrophage-like cells, suggesting that blockade of the p38 MAPK pathway may represent a therapeutic approach to treat discogenic pain.^137^ Zhang et al. demonstrated that TGF-β1 significantly decreases CCL4 expression by activating ERK1/2 MAPK signaling in NP cells and prevents disc degeneration.^131^ Interestingly, these researchers also found that TGF-β1 alleviates inflammatory responses in the DRG and relieves pain behaviors in a rat model.^131^ This study indicated that the TGF-β1 and ERK1/2 MAPK signaling pathways may serve as therapeutic targets for the cure of inflammation-related pain associated with IVDD.^131^

### TLR signaling

TLRs, an integral part of the innate immune system, are activated by pathogen-associated molecular patterns, such as bacterial cell wall debris.^138^ It has been reported that TLRs 1, 2, 4, and 6 are expressed by disc cells and correlate with the severity of disc degeneration.^139^ Krock et al. found that TLR2 activation induces NGF and BDNF gene expression and NGF protein secretion in human IVD cells, which could be used to target NGF to treat LBP associated with disc degeneration.^132^ Recently, the same group showed that chronic TLR4 inhibition alleviated behavioral signs of LBP, pain-related neuroplasticity, and disc inflammation in SPARC-null mice.^41^ In addition, TAK-242 inhibits TLR4 activation in the IVDs and significantly reduces cytokine release.^41^ Li et al. showed that resistin bound directly to TLR4 and increased CCL4 expression in NP cells via the p38-MAPK and NF-κB pathways, which ultimately led to macrophage infiltration.^140^ It has also been reported that high mechanical loading not only promotes the secretion of inflammatory and pain-related factors of IVD cells, such as TNF-α, IL-6, IL-8, IL-17, IFN-γ, monocyte chemoattractant protein 1 (MCP-1), and NGF, but also upregulates TLR2 and TLR4 expression, indicating that mechanical stress may play important roles in inducing and maintaining discogenic LBP.^141,142^ Therefore, TLRs are potential therapeutic targets to cure disc degeneration and reduce discogenic pain.

## Molecules under laboratory investigation to target inflammation and discogenic pain

**Fig. 4:**
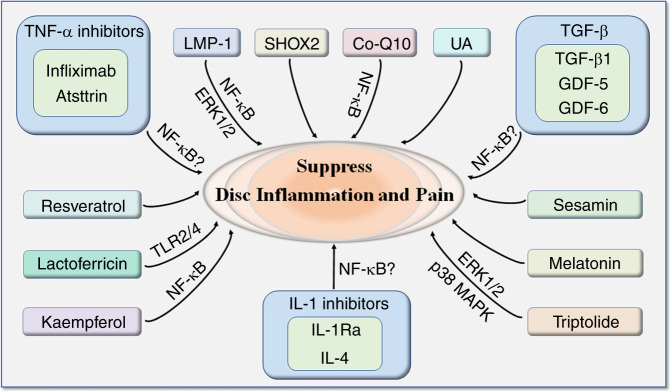
Molecules under laboratory investigation to target inflammation and discogenic pain. IVD intervertebral disc, TNF-α tumor necrosis factor α, LMP-1 LIM mineralization protein-1, SHOX2 short stature homeobox 2, TGF-β transforming growth factor β, GDF growth and differentiation factor, IL-1Ra IL-1 receptor antagonist

Since inflammation plays a vital role in the production of discogenic LBP, various molecules, and chemicals have been investigated by researchers worldwide for their potential to suppress inflammation and related discogenic pain, as summarized in Fig. 4.

### TNF-α inhibitors

TNF-α is an important cytokine involved in inflammatory signaling. TNF-α inhibitors, including infliximab and atsttrin, have been shown to suppress inflammation and pain. Infliximab is an anti-TNF-α antibody. When injected intradiscally in rats, infliximab reduced pain to sham levels.^39^ Atsttrin is a synthesized protein containing three fragments of progranulin, a growth factor implicated in inflammation. This protein can bound directly to TNF-α receptors and antagonized TNF-α-initiated inflammatory signaling in a mouse model of multiple arthritis.^143^ In ex vivo cultured murine and human discs, atsttrin also decreased TNF-α-triggered inflammatory cytokine production (MMP-13, COX-2, iNOS, and IL-17) and subsequent catabolic changes, including loss of cartilage, disc height, and NP cells.^86^ Due to its long half-life, high efficacy, low molecular weight and no oncogenic effect over progranulin, atsttrin warrants further investigation in the management of inflammatory IDD.

### IL-1 inhibitors

IL-1 receptor antagonist (IL-1Ra) is a natural inhibitor of IL-1. This molecule binds to the IL-1 receptor (IL-1R), thus preventing the binding of IL-1 to IL-1R and the delivery of inflammatory signals. IL-1Ra knockout in mice led to accelerated IDD, represented by decreased proteoglycan, disrupted collagen structure, increased matrix-degrading enzymes, including MMP3, MMP7, and ADAMTS4,^144^ and a higher histological grade of degeneration. IL-1Ra-deficient IVD cells also exhibited decreased proliferation.^144^ Further evidence showed that IL-1Ra might have a therapeutic effect on discogenic pain. Treatment of nondegenerated or degenerated human IVD explants with IL-1Ra reversed the catabolic effect of IL-1, including the expression of matrix degradation proteases, including type II collagenase, gelatinase, caseinase, and MMP3. Moreover, a single injection of IVD cells overexpressing IL-1Ra in the explants caused a significant inhibition of the expression of all matrix degradation enzymes, which could be sustained for 2 weeks.^101^

IL-4 is an anti-inflammatory cytokine. In macrophages, IL-4 inhibits the production of TNF-α and IL-1β and induces the secretion of IL-12.^145^ In DRGs, overexpression of IL-4^146^ suppressed pain-related behaviors, including mechanical allodynia and thermal hyperalgesia, and decreased c-Fos, a histologic marker of nociceptive processing in the dorsal horn. In addition, the upregulation of inflammatory cytokines, including PGE2 and IL-1β, was retarded.^146^ In IVD cells, IL-4 treatment reduced inflammatory gene expression of IFN-beta, IL-12, IL-6, and IL-8 and downregulated the protein release of IL-6 and IL-8 in LPS-treated IVD cells.^147^

### Transforming growth factor beta (TGF-β) superfamily

TGF-β is a group of proteins involved in the early regulation of cell growth and development. Among them, TGF-β1, growth and differentiation factor 5 (GDF-5) and GDF-6 have been shown to suppress inflammation in the IVD.

TGF-β1 can downregulate TNF-α expression induced by IFN-γ and IL-1β and antagonize collagen I and MMP3 expression induced by TNF-α in NP cells.^148^ An in vivo study showed that intradiscal injection of TGF-β1 downregulated CCL4 expression, prevented the inflammatory response and reduced degenerative features and pain behavior in rats with induced IDD.^131^ In a rat model of neuropathy, intrathecal infusion of TGF-β1 significantly attenuated the development of pain hypersensitivity and reversed previously established pain.^149^ The effect of TGF-β is reported to be exerted through ERK1/2 signaling, which could be abolished by specific inhibitors.^148^

GDF-5 is a protein involved in skeletal and joint development. GDF-5 gene polymorphism is associated with IDD susceptibility.^150^ GDF-5 deficiency in mice resulted in decreased proteoglycan and collagen II levels and abnormal histology in the IVD.^151^ In rat NP cells,^152^ GDF‑5 overexpression inhibited the secretion of TNF‑α, IL‑1β, PGE2, and NO in culture medium induced by LPS, inhibited the decrease in matrix production, including collagen II and aggrecan, and prevented NF‑κB activation induced by LPS.

GDF-6 (BMP13) has an important role in early embryonic and spinal column development.^153^ Injection of GDF-6 in the early stages of IDD can prevent the loss of ECM proteins and retain greater hydration and cells in the NP.^154^ Clarke et al. found that GDF-6 can stimulate adipose-derived MSCs (AD-MSCs) to differentiate into NP-like cells. The differentiated AD-MSCs show an increase in aggrecan-to-type II collagen gene expression.^155^ GDF-6 also has the potential to enhance ECM accumulation and induce cell migration in certain disc cells.^156^ Miyazaki et al. found that the expression of TNF-α, IL-6, VEGF, COX-2, and NGF was significantly reduced by injecting GDF-6 into a rabbit puncture model of IVD. GDF-6 injection had a certain effect on IDD and attenuated degenerated IVD-induced pain.^157,158^

### LIM mineralization protein-1 (LMP-1)

LMP-1 regulates osteogenesis and chondrogenesis. Overexpression of LMP-1 increased, whereas knockdown of LMP-1 decreased, the production of ECM components, including collagen II, aggrecan, versican, and soluble GAG,^159,160^ via ERK1/2 activation.^160^ Moreover, overexpression of LMP-1 abolished TNF-α-mediated MMP-3 and MMP-13 expression by inhibiting p65 translocation, as well as MMP-3 and MMP-13 promoter activity.^160^ These results indicated that LMP-1 has an anti-inflammatory effect against TNF-α to maintain IVD possibly via ERK1/2 activation and NF-κB inhibition.

### Short stature homeobox 2 (SHOX2)

SHOX2 is a member of the short stature homeobox gene family and is essential in skeletal development. In vivo human IDD or in vitro TNF-α treatment led to decreased expression of SHOX2.^161^ Treatment with SHOX2 siRNA inhibited the proliferation and apoptosis of NP cells, decreased aggrecan and collagen II expression, and increased MMP3 and ADAMTS-5 production in NP cells.^161^ These results indicated that SHOX2 has a protective effect in the IVD and is worth further investigation.

### Melatonin

Melatonin is a natural hormone in the body involved in biorhythm regulation. In the IVD, melatonin diminished cellular apoptosis caused by tert-butyl hydroperoxide, maintained ECM production, and suppressed the expression of degenerative enzymes, including MMP-13 and ADAMTS-5, in NP cells.^162^ The effect of melatonin is partially related to its activation of autophagy and mitophagy. Melatonin activated Parkin, the upstream regulator of mitophagy, in a dose and time-dependent manner in NP cells. Mitophagy inhibition by cyclosporine A partially eliminated the protective effects of melatonin.^162^ In vitro cell culture and in vivo rat model study showed that melatonin delayed the progression of IL-1β-induced IVDD and related LBP via inhibiting the IL-1β/NF-κB-NLRP3 inflammasome activation positive feedback loop, and downregulating mitochondrial reactive oxygen species (mtROS) production, suggesting melatonin might be a considerable potential clinical agent for IVDD.^163^

### Resveratrol

Resveratrol is a polyphenolic phytoalexin in red wine with an antioxidative effect. Dietary supplementation of grape polyphenol, which contained resveratrol, to rats with punctured discs reduced the behavioral sensitivity and gene expression levels of proinflammatory cytokines in DRGs but failed to alleviate disc degeneration or change the proinflammatory cytokine level in IVD.^164^ In vitro, resveratrol partially counteracted the inflammatory effects of TNF-α and IL-1β, including decreased ECM content and increased NP cell senescence and matrix degradation enzymes (MMP-3, MMP-13, and ADAMTS-4).^165^ Resveratrol also showed a similar anticatabolic effect to reverse the matrix degradation and apoptotic induction of oxidative damage caused by hydrogen peroxide in NP cells.^166^ The effect may be mediated by the activation of autophagy^165^ through the PI3K/Akt pathway^167^ or AMPK/SIRT1 signaling pathway^168^ but does not seem to involve the MAP kinase pathways or the NF-κB/SIRT1 pathway.^169^ Furthermore, in a rodent model, resveratrol ameliorated pain behavior triggered by the application of NP tissue to the DRG.^169^ Overall, resveratrol seems to have a promising effect on the treatment of IVD-derived pain.

### Triptolide

Triptolide is a natural substance found in the Chinese medicinal herb *Tripterygium wilfordii* Hook. A recent study^170^ demonstrated that triptolide at low concentrations (50 nmol·L^–1^) had an anti-inflammatory effect in human IVD cells by suppressing the expression of IL-6/-8, PGE2S, MMP1/2/3/13, and TLR2/4 and an anticatabolic effect on the production of aggrecan and collagen-II. However, a higher concentration of triptolide resulted in an upregulation of TNF-α, indicating its adverse effect at the high dose. It was further found that these effects of triptolide were achieved via the MAP kinases p38 and ERK but not via the JNK or NF-κB pathways.^170^

### Kaempferol

Kaempferol is a natural flavonol found in many plants. In bone marrow MSCs, kaempferol decreased MMP3 and MMP13, alleviated LPS-induced inflammation by reducing the level of the proinflammatory cytokine IL-6 and increasing anti-inflammatory cytokines such as IL-10 by inhibiting the nuclear translocation of NF-κB p65.^171^ However, since in the same study,^171^ kaempferol was shown to inhibit chondrogenesis while promoting the osteogenesis of bone marrow MSCs, a question might be raised regarding whether the application of kaempferol with MSCs may increase the risk of ectopic bone generation in IVDs.

### Sesamin

Sesamin is a bioactive component extracted from sesame. Li et al.^172^ tested the effect of sesamin on LPS-induced IDD. These researchers reported that sesamin inhibited the activation of LPS-induced JNK but not p38 or ERK. As a result, this molecule effectively inhibited LPS-induced inflammatory factors (IL-1β, TNF-α, iNOS, NO, COX-2, and PGE2) and the production of catabolic enzymes (MMP-1, MMP-3, MMP-13, ADAMTS-4, and ADAMTS-5) in a dose-dependent manner in rat disc explants. Sesamin also blocked the LPS-induced migration of macrophages and the degradation of collagen II and aggrecan.

### Lactoferricin

Lactoferricin is an amphipathic, cationic peptide derived from milk protein. Bovine lactoferricin has been shown to significantly attenuate the IL-1β and LPS-mediated suppression of proteoglycan production in human and bovine NP cells.^173^ Simultaneously, lactoferricin reduced multiple degrading enzymes, including MMP-1, MMP-3, MMP-13, ADAMTS-4, and ADAMTS-5, in bovine NP cells.^173^ In addition, lactoferricin suppresses oxidative and inflammatory factors, such as iNOS, IL-6, Toll-like receptor-2 (TLR-2), and TLR-4.^173^

### Coenzyme Q10 (Co-Q10)

Co-Q10 is a coenzyme that plays a vital role in the electron transport chain. A recent study by Wang et al.^174^ suggested that Co-Q might have anti-inflammatory effects on IVDs. In IL-1β-treated human NP cells, the expression of inflammatory biomarkers, including TNF-α, COX-2, IL-6, and iNOS, was reduced by Co-Q10. Co-Q10 also helped to prevent the IL-1β-induced reduction in collagen 2, aggrecan, and Sox-9. It was further postulated that the anti-inflammatory effect potentially occurs through the inhibition of NF-κB signaling activation, while the anabolic impact of Co-Q10 is possibly associated with the Akt activation signaling pathway.

### Urolithin A (UA)

Urolithin A (UA) is a metabolite transformed from ellagitannins by gut bacteria. Recent studies have indicated UA has anti-inflammatory and antioxidant properties.^175,176^ A 2018 study^177^ investigated the effect of UA treatment on IDD. UA ameliorated hydrogen peroxide-induced cell senescence and decreased the TNF-α-induced reduction in collagen II and the production of MMP3 and MMP13 in NP cells. In a rat tail model, UA treatment alleviated the puncture-induced reduction in disc height, the increase in Pfirrmann grade scores and disc histological destruction. It was further revealed that UA inhibited ERK, JNK, and Akt phosphorylation but had no influence on the NF-κB p65 and p38-MAPK pathways.

In summary, LMP-1, kaempferol, Co-Q10, and possibly TNF-α inhibitors and IL-1 inhibitors exert their effects by inhibiting NF-κB signaling. TGF-β may also belong to this group, since TGF-β can inhibit TNF-α and IL-1β secretion. LMP-1, triptolide and UA can mediate their effects through ERK1/2 activation, while lactoferricin suppresses TLR2/4. Triptolide also deactivates p38-MAPK. The majority of the above molecules showed anti-inflammatory effects and prevented disc matrix degradation. However, only a few of them, including TNF-α inhibitors, IL-1 inhibitors, TGF-β1, GDF-6 and resveratrol, are effective in alleviating discogenic pain. The other discussed molecules can suppress inflammation, but whether they can regulate pain production awaits further study.

## Clinical treatments and trials suppressing inflammation to treat discogenic LBP

In this section, we focus on bioactive agents, which exert their pain-alleviating effects by suppressing inflammation, being tested in clinical trials.

### TNF-α inhibitors

The clinical effect of TNF-α inhibitors on discogenic LBP has not been reported but has been investigated in disc-related diseases, such as sciatica (Table 2). In a clinical trial of treating severe sciatica with infliximab, Karppinen et al.^178^ found that the pain was significantly relieved at 1 h after the injection, and there was no need for surgery at 3 months of follow-up. Cohen et al.^179^ performed a double-blinded trial to investigate epidural etanercept, an anti-TNF-α medication, in the treatment of sciatica. One month after treatment, etanercept delivered significant improvements in leg and back pain, with 17% in the saline group, 100% in the 2 mg group, and 67% each in the 4 mg and 6 mg groups reporting at least a 50% reduction in leg pain and a positive global perceived effect, which persisted for 6 months after treatment. Currently, a clinical trial investigating the effect of infliximab in treating chronic LBP and modic changes is recruiting patients.^180^

**Table 2:** Biological treatments under clinical development to treat discogenic LBP

Therapeutics	Authors	Year	Number of patients	Study design	Treatment	Analysis variables	Follow-up period	Outcomes
PRP	Akeda et al.^183^	2011	6	Prospective single arm	Intradiscal injection of 1.5 mL of PRP	VAS, RDQ, MRI (T2)	6 M	VAS and RDQ were decreased at 1 month and sustained for 6 months. No change in T2 values was observed.
PRP	Tuakli-Wosornu et al.^186^	2016	47	Prospective double-blind RCT	Intradiscal injection of 1–2 mL of PRP (*n* = 29) vs. contrast agent (*n* = 18)	FRI, NRS, 36-item Short Form Health Survey, and modified NASS Outcome Questionnaire	12 M	The improvement of patients’ LBP symptoms and function occurred as early as 8 weeks after treatment and was maintained for at least 1 year.
PRP	Levi et al.^185^	2016	22	Single arm	Intradiscal injection of 1.5 mL of PRP at one or more levels	VAS, ODI	6 M	14%, 32%, and 47% of the patients achieved a successful outcome at 1, 2, and 6 months, while the percentages reaching a 50% decrease in VAS were 36%, 41%, and 47%.
PRP	Lutz^187^	2017	1	Case report	Intradiscal injection of 1.5 mL of PRP	Pain, range of motion, MRI (T2)	12 M	Improvements in pain and range of motion and increased T2 nuclear signal intensity were observed.
PRP	Akeda et al.^184^	2017	14	Prospective single arm	Intradiscal injection of 2 mL of PRP	VAS, RDQ, X-ray, MRI (T2)	12 M	The mean pain scores before treatment (VAS: 7.5 ± 1.3; RDQ: 12.6 ± 4.1) were decreased at 1 month and were sustained at 6 months (VAS, 3.2 ± 2.4, RDQ; 3.6 ± 4.5) and 12 months (VAS, 2.9 ± 2.8; RDQ, 2.8 ± 3.9) after treatment. No significant changes in the mean T2 values was observed.
TNF-α inhibitors	Karppinen et al.^178^	2003	72	Open-label, controlled study to treat sciatica	Infliximab (3 mg, *n* = 10) vs. saline (*n* = 62)	VAS, ODQ, MRI (T2 and T1), SLR, Schober	3 M	The infliximab group had more pain reduction than the control group (painless patients after 2 weeks: 60% vs. 16%; after 3 months: 90% vs. 46%). At 1 month, all patients in the infliximab group went back to work, while 38% in the control group remained on sick leave.
TNF-α inhibitors	Cohen et al.^179^	2009	24	Double-blind, controlled, multidose	Epidural etanercept (*n* = 18) vs. placebo (*n* = 6)	NRS, ODI, global perceived effect	1 M	Improvements in leg and back pain were reported in the treated group after 1 month. One patient in the saline group (17%), six patients in the 2 mg group (100%), and four patients each in the 4 mg and 6 mg groups (67%) reported >50% reduction in leg pain and a positive global perceived effect 1 month after treatment, which persisted to 6 months after treatment.
TNF-α inhibitors	–	2018	126	RCT double blind, multicenter	Infliximab vs. placebo	ODI, NRS, STIR, RDQ	9 M	Recruiting (Clinical trial ID: NCT03704363)
NGF inhibitors	Katz et al.^189^	2011	217	RCT double-blind, multicenter, parallel	Tanezumab (*n* = 88), naproxen (*n* = 88) or placebo (*n* = 41)	aLBPI, RDQ, BPI-SF, PGA, Patients’ Global Evaluation, rescue medication use	6 W	The tanezumab group had a better reduction in aLBPI, RDQ and other secondary outcomes, except rescue medication use, than the naproxen or placebo group.
NGF inhibitors	Kivitz et al.^190^	2013	1 347	RCT, double-blind, multicenter, parallel phase IIB	Tanezumab (5, 10, or 20 mg), naproxen (500 mg), or placebo	aLBPI, RDQ, PGA	16 W	Tanezumab at 10 and 20 mg had a similar efficacy in improving aLBPI, RDQ, and the PGA scores vs. both placebo and naproxen. Tanezumab at 5 mg improved the PGA scores vs. placebo. Arthralgia, pain in extremity, headache, and paresthesia were the most commonly reported AEs by tanezumab.
NGF inhibitors	Gimbel et al.^191^	2014	848	Noncontrolled, randomized, multicenter	Tanezumab at 10 mg (*n* = 321) vs. 20 mg (*n* = 527)	BPI-SF, RDQ, PGA	200 d	Both tanezumab at 10 mg and 20 mg provided sustained effectiveness. Tanezumab at 10 mg had better tolerability.
NGF inhibitors	Hochberg et al.^192^	2016	1 564	Blinded adjudication of previous reports	Tanezumab	–	~200 d	Tanezumab treatment was not associated with an increase in osteonecrosis but was associated with an increase in rapid progression of osteonecrosis.

*NRS* numeric rating scale, *VAS* visual analog scale, *ODI* Oswestry disability index, *RDQ* Roland-Morris disability questionnaire, *FRI* functional rating index, *RCT* randomized controlled trial, *aLBPI* average LBP intensity, *STIR* short tau inversion recovery, *PGA* patient’s global assessment, *AEs* adverse events, *NASS* North American Spine Society, *SLR* straight-leg-raising test, *BPI-SF* brief pain inventory-short form

### Platelet-rich plasma (PRP)

PRP has been reported to alleviate inflammation and pain in various systems.^181,182^ PRP contains a cocktail of growth factors, such as platelet-derived growth factor, TGF-β, epidermal growth factor, insulin-like growth factor 1 and vascular endothelial growth factor. It also contains blood-clotting factors and proteinase inhibitors. It is possible that the complex ingredients in PRP together exert multiple effects to dampen inflammation and promote tissue regeneration. For example, we know from our previous analysis that TGF-β plays a role in suppressing painful disc degeneration.

PRP has been clinically investigated as a biologic therapy to stimulate disc regeneration or at least delay disc degeneration (Table 2). In 2011, Akeda et al. injected six LBP patients with 2 mL of PRP.^183^ The results showed that the mean pain scores decreased from 7.1 to 1.8 on a numeric rating scale (NRS) at 1 month, and this effect was maintained at 6 months, although there was no change in mean T2 values. Later, the same group injected 14 LBP patients with 2 mL of PRP.^184^ The results showed that the mean pain scores on a NRS decreased from 7.5 at 1 month to 2.9 at 12 months, and the RDQ decreased from 12.6 at 1 month to 2.8 at 12 months.^184^ In another clinical study, Levi et al.^185^ studied the safety and feasibility of intravertebral disc injection of PRP to treat disc degeneration. The study included 22 patients with disc herniation. Each treated disc received an injection of 1.5 mL of PRP into the center of the NP under dioptric guidance. An unqualified success was defined as at least a 50% improvement in the VAS and a 30% decrease in the ODI. A total of 14%, 32%, and 47% of the patients achieved a successful outcome at 1, 2, and 6 months, respectively, while the percentages reaching a 50% decrease in VAS were 36%, 41%, and 47%, respectively, demonstrating a satisfactory effect. In 2016, a prospective randomized controlled trial enrolled 47 patients for the application of PRP to treat discogenic LBP.^186^ Twenty-nine patients received PRP injection, while 18 patients received contrast agent only. The improvement of patients’ LBP symptoms and function occurred as early as 8 weeks after treatment, and the effect could be maintained for at least one year. No adverse events were observed. In 2017, Lutz^187^ reported a clinical case in which intradiscal injection of 1.5 mL of autologous PRP improved the symptoms of an LBP patient who returned to athletic activities as early as 6 weeks after the surgery. This case is the only one to show improved MRI signals by PRP injection.

In summary, the current studies consistently supported the safety and efficiency of PRP treatment in relieving disc pain and function.

### NGF inhibitors

NGF regulates painful nerve activity and plays a vital role in pain signaling pathways.^188^ Katz et al.^189^ compared the remission of symptoms in patients with chronic nonradiculopathic pain treated with tanezumab, a monoclonal antibody with a high affinity for NGF, by intravenous injection. In total, 88 patients received tanezumab (200 μg·kg^–1^), 88 patients received placebo plus oral naproxen 500 mg twice a day, and 41 patients received placebo. After 6 weeks, the patients receiving tanezumab experienced a significant reduction in LBP compared to that of the other two groups. Tanezumab caused no serious adverse events but temporal and mild adverse events of abnormal peripheral sensation. Another follow-up trial with 1 347 patients^190^ investigated the long-term safety and efficacy of different doses of tanezumab for chronic LBP and reported that tanezumab at 10 and 20 mg had a similar effect in resolving pain and disability. However, tanezumab at various doses caused higher adverse events than placebo or naproxen. These events included arthralgia, pain in extremity, headache, and paresthesia, which resulted in the temporary termination of this trial.^190^ Later, an extension report from the same group^191^ included 321 patients for the 10 mg dose and 527 patients for the 20 mg dose administered intravenously for an average 200 days at 8-week intervals. Both doses showed a similar effect in LBP relief. The majority of adverse effects included arthralgia, paresthesia, and hypoesthesia. Osteonecrosis was also observed. It was concluded that the 10 mg dose was better tolerated than the 20 mg dose. However, blinded adjudication of the above studies^192^ revealed that osteonecrosis was not primary in these cases, but tanezumab treatment was associated with an increase in the rapid progression of osteoarthritis.

Overall, it was concluded from the above studies that treatment with 10 mg tanezumab met the primary endpoint, demonstrating a significant improvement in pain at 16 weeks compared to placebo, and the adverse reactions were not severe.

## Discussion

Disc degeneration is a common phenomenon in the aged population and has a complex etiology. Painful disc degeneration is a major contribution to LBP, during which persistent inflammation is thought to be an important factor.^8,15^ In this review, we focused on the contribution of inflammation to disc degeneration and the associated LBP. We reviewed laboratory studies on potential candidate molecules to treat disc degeneration by dampening inflammation and summarized the current progress of clinical trials targeting inflammation and discogenic pain.

Among the laboratory studies on IVD regeneration, we focused on those that investigated the effect of the target molecule on suppressing IVD inflammation and pain. These anti-inflammatory molecule candidates include TNF-α inhibitors, IL-1Ra, IL-4, TGF-β1, LMP-1, SHOX2, GDF-6, lactoferricin, triptolide, kaempferol, Co-Q10, UA, and sesamin, with a focus on the inhibition of TNF-α and IL-1β. These molecules all have the potential for future drug development to treat painful disc degeneration, providing a full understanding of their effect and underlying mechanism. However, among these, only TNF-α inhibitors, IL-1 inhibitors, TGF-β1, GDF-6, and resveratrol have been demonstrated to be effective in alleviating discogenic pain. The effect of the others on pain suppression awaits further study.

Despite showing potential in laboratory tests, many anti-inflammatory molecules explored in the laboratory, as mentioned above, have not yet been used in clinical investigation, possibly because only a few laboratory studies have been conducted on these molecules. Therefore, there are insufficient exploratory and repeated studies to support a wide recognition of these molecules for their potential in painful disc treatment. Further studies, which assess safety and efficacy, will be necessary before these molecules can be used as drugs. To date, the potential therapies under clinical investigation to treat LBP include TNF-α inhibitors, PRP, and NGF inhibitors. These reagents have unique advantages in clinical development in that they are established in the treatment of other diseases. The delivery methods include intravertebral disc injection or intravenous infusion. The majority of these trials demonstrated promising efficacy in alleviating pain and restoring spinal motion. However, since the follow-up time is limited, it is not clear whether these treatments are effective for a long period of time. In addition, most trials focused on pain assessment without assessing or showing any effect on the biological repair of IVDs, and only one trial among these detected IVD restorations, as represented by an improved MRI T2 signal. Therefore, evidence of these drugs in IVD repair is still lacking, and it is also unclear whether the treatment affects the natural process of IDD. More importantly, although the timing of the intervention has not been investigated in these studies, this factor might have a significant impact on the outcome of the treatment, as less degenerated discs could be easier to regenerate, while severely deranged discs are hard to repair.^193^ In addition, psychological factors may play a role in some unexplained back pain, which makes it difficult to assess and identify appropriate patients for treatment.

Currently, the majority of studies on IVD have focused on NP and AF with less emphasis on EP and subchondral bone.^75,194^ However, the IVD is an integrated tissue with three parts closely interacting with each other to constitute the structure, function and metabolism. The EP seals the NP and contains the main diffusion channel of nutrients into the IVD, and EP damage or calcification can alter the biomechanical behaviors, pattern and nutrient supply as a pain generator or accelerator of IDD. Few studies have explored the degeneration of EP and subchondral bone and reported its association with pain introduction. Encouragingly, some recent studies have taken one step forward. For example, Lv et al.,^195^ Bailey et al.,^196^ and Munir et al.^197^ independently reported that the grade of EP defects positively correlated with the grade of IDD or chronic LBP. More studies are needed in this field. More importantly, treating IVD as a whole instead of three isolated sections may be the future direction and provide novel insights into the treatment of painful IVD.
